# Proteomic Analysis of Serum Proteins from Patients with Severe Coronary Artery Calcification

**DOI:** 10.31083/j.rcm2307229

**Published:** 2022-06-24

**Authors:** BuChun Zhang, XiangYong Kong, GuangQuan Qiu, LongWei Li, LiKun Ma

**Affiliations:** ^1^Department of Cardiology, The First Affiliated Hospital of USTC, Division of Life Sciences and Medicine, University of Science and Technology of China, 230001 Hefei, Anhui, China

**Keywords:** coronary calcification, data-independent acquisition, quantitative proteomics, serum, atherosclerosis

## Abstract

**Background:**

Proteomic studies investigating novel molecular markers of coronary artery calcification (CAC) are scarce.This study compared the protein expression in the serum of patients with severe CAC and non-CAC.

**Methods:**

The serum from 30 patients with severe CAC and 30 matched-controls were screened by data-independent acquisition(DIA)-based proteomic technology. Bioinformatics analysis tools were used to analyze the underlying molecular mechanisms of the differentially expressed proteins. Candidate proteins were further validated by an enzyme-linked immunosorbent assay (ELISA) in an independent cohort. A receiver operating characteristic (ROC) curve was used to estimate the diagnostic power of the candidate proteins.

**Results:**

Among the 110 identified proteins, the expression of 81 was significantly upregulated, whereas 29 proteins were downregulated (fold change ≥1.5; *p *< 0.05) between patients with and without CAC. Bioinformatics analysis indicated that the differential proteins are involved in complement and coagulation cascades, platelet activation, regulation of actin cytoskeleton, or glycolysis/gluconeogenesis pathways. Further verification showed that serum levels of complement C5 (C5), fibrinogen gamma (FGG), pyruvate kinase isoform M2 (PKM2), and tropomyosin 4 (TPM4) were consistent with the proteomic findings, which could allow discrimination between CAC and non-CAC patients.

**Conclusions:**

This study revealed that high serum levels of serum C5, FGG, PKM2, and TPM4 proteins were linked to severe CAC. These proteins may be developed as biomarkers to predict coronary calcification.

## 1. Introduction

Coronary artery calcification (CAC) is a hallmark of advanced atherosclerosis and a well-established predictor of future cardiovascular events [1, 2]. The presence and extent of CAC can be assessed by traditional coronary angiography, intravascular ultrasound (IVUS), optical coherence tomography (OCT) and coronary computed tomographic angiography (CTA) [3]. Although vascular calcification was previously considered passive and degenerative, recent studies have suggested that it is an active process stimulated by inflammatory pathways [4]. Some risk factors for coronary disease including dyslipidemia and diabetes are correlated with the development of calcification [5]. However, long-term statin therapy and adequate diabetes control fail to stop the calcification process [6], indicating potential molecular mechanisms for calcification other than the conventional risk factors of atherosclerosis. The pathomechanism of vascular calcification is still obscure, therefore a greater understanding of the molecular mechanisms underlying coronary calcification could help to seek new intervention targets. Therefore, further elucidation of the dysregulated biological pathways and identification of novel diagnostic biomarkers specific for coronary calcification are needed.

Serum proteomic analysis is an important tool for disease biomarker discovery and provide information on the underlying pathological processes, possibly superior to genome or transcriptome [7]. Compared with traditional data-dependent acquisition (DDA)-based proteomics research, data-independent acquisition (DIA) is a powerful label-free technique in proteomic studies with high quantitative accuracy and reproducibility [8, 9]. However, studies using DIA technology to investigate the serum proteome profile for CAC patients are lacking.

In this study, we first utilized DIA proteomic techniques to identify serum proteins closely associated with CAC in the discovery phase. Differentially expressed proteins (DEPs) were further validated via an enzyme-linked immunosorbent assay (ELISA) approach in an independent sample. This study not only identified potential protein biomarkers for CAC diagnosis but also provides insights into the pathophysiological mechanisms of CAC.

## 2. Materials and Methods 

### 2.1 Study Design and Participants

**Fig. 1.**
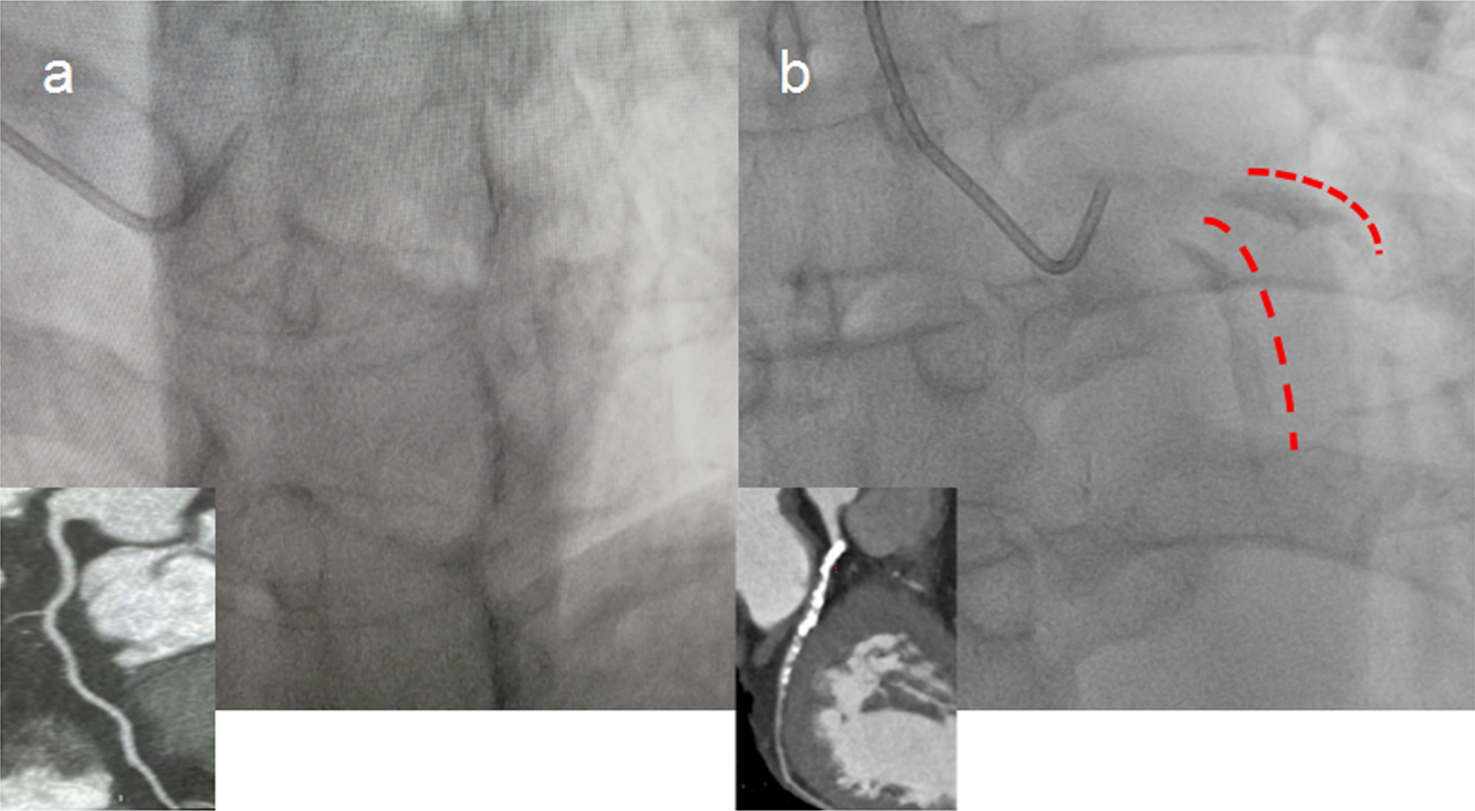
**Representative image of severe calcified coronary lesions detected by coronary angiography**. (a) Controls without coronary artery calcification (non-CAC). (b) Patients with coronary artery calcification (CAC). Red marks indicate severe calcified left anterior descending artery (LAD) lesions detected by x-ray and the corresponding image from coronary computed tomography angiography (CTA).

Patients in this study were from our prospective registration study, in which patients with severe calcified coronary lesions on angiographic images who underwent percutaneous coronary intervention (PCI) with orbital atherectomy were recruited between February 2020 and May 2021 (registered on the Chinese Clinical Trial Registry [ChiCTR] 2000029775). Severe calcified coronary lesions were defined as the presence of apparent radiative opacities within the coronary lumen [10]. Based on qualitative visual assessment of calcified lesions using fluoroscopy, they were classified as severe (radio-opacities located on both sides of the coronary arterial wall before contrast injection and without cardiac movement), moderate (densities were acquired during the cardiac cycle before contrast injection), or none/mild calcified lesions according to the published literature [11]. An example of severe calcified coronary lesions on angiographic images is given in Fig. 1. Two cardiologists who were unaware of the patients included in this study assessed the angiographic calcification. Some cases of disagreement were adjudicated by a third independent cardiologist and given the final diagnosis. The recruited control subjects were matched by age, sex, and conventional risk factors. The control subjects were selected during the same period in the same hospital with non-calcific coronary angiography findings. Subjects with a known history of malignant tumors, serious system diseases, severe respiratory disease, renal or hepatic insufficiency, and acute infections were not included. The clinical and demographic information of the participants was recorded. The study protocol was approved by our institutional ethics committee, and written informed consent was obtained from all patients. The study was designed in accordance with the ethical guidelines of the Declaration of Helsinki.

### 2.2 Serum Sample Collection for Proteomics Analysis

Thirty individuals were randomly selected from each group and subjected to proteomics analysis in a discovery setting. Fasting venous blood samples were collected preoperatively from CAC and control patients, centrifuged at 2500 g for 10 min to obtain serum, collected in EDTA-coated tubes, and stored at –80 °C until subsequent analysis.

### 2.3 Sample Preparation and Data Dependent Acquisition (DDA) Library Generation 

High-abundant proteins from 20 μL of each serum sample were removed using the Human 14 Multiple Affinity Removal Column (4.6 × 50 mm, Agilent Technologies, USA) according to the manufacturer’s protocol. The high- and low-abundance proteins were collected, and a 5 kDa ultrafiltration tube (Sartorius, Göttingen, Germany) was used for desalination and concentration of the high- and low-abundance components. SDT buffer (4% SDS, 100 mM DTT, 150 mM Tris-HCl pH 8.0) was added, boiled for 15 min, and centrifuged at 14,000 g for 20 min. The supernatant was quantified with the BCA Protein Assay Kit (Bio-Rad, Hercules, CA, USA). The sample was stored at –80 °C.

### 2.4 Filter-Aided Sample Preparation (FASP) Digestion Procedure

Both high- and low-abundant proteins were subjected to a digestion procedure modified from the filter-aided sample preparation (FASP) protocol as previously described [12]. Briefly, 200 μg protein was mixed with 30 μL SDT buffer (4% SDS, 100 mM DTT, 150 mM Tris-HCl pH 8.0). UA buffer (8M urea, 150 mM Tris HCl pH 8.0) accompanied by repeated ultrafiltration (Microcon-10 kDa Centrifugal Filter Unit; Millipore, Burlington, MA, USA) removed the detergent, DTT, and other low-molecular-weight components. Then 100 μL iodoacetamide (IAA) (100 mM IAA in UA buffer) was added to the samples and stored in the dark for 30 min at 4 °C. Subsequently, 100 μL UA buffer washed the filters three times and then twice with 100 μL 25 mM ${\mathrm{NH}}_{4}$${\mathrm{HCO}}_{3}$. Next, 40 μL trypsin buffer (2 μg trypsin in 40 μL of 25 mM NH4HCO3 buffer) was added to the samples for trypsin digestion. Then the peptides of each sample were desalted on the C18 cartridge column (Empore™ SPE Cartridges C18 (standard density), bed I.D. 7 mm, volume 3 mL; Sigma, St. Louis, MO, USA) and dried under a vacuum. The peptide concentration was estimated by ultraviolet light spectral density at 280 nm using an extinction coefficient of 1.1 of 0.1% (g/L) solution, which was calculated based on the frequency of tryptophan and tyrosine in vertebrate proteins.

### 2.5 Data-Dependent Acquisition (DDA) Mass Spectrometry Analysis

All fractions for data-dependent acquisition (DDA) library generation were analyzed by the Thermo Scientific Q Exactive HF X Mass Spectrometer connected to the Easy-nLC 1200 Chromatography System (Thermo Fisher Scientific, Waltham, MA, USA). The peptide (1.5 μg) was first loaded onto the EASY-SprayTM C18 Trap Column (P/N 164946, 3 μm, 75 μm × 2 cm; Thermo Fisher Scientific) and then separated on the EASY-SprayTM C18 LC Analytical Column (ES802, 2 μm, 75 μm × 25 cm; Thermo Fisher Scientific) with a linear gradient of buffer B (84% acetonitrile and 0.1% formic acid) at a flow rate of 250 nL/min over 120 min. The mass spectrometry (MS) detection method was positive ion, the scan range was 300–1800 m/z, the resolution for the MS1 scan was 60,000 at 200 m/z, automatic gain control (AGC) was 3e6, maximum IT was 25 ms, and dynamic exclusion was 30.0 s. Each full MS-SIM scan followed 20 ddMS2 scans. The resolution for the MS2 scan was 15,000, the AGC target was 5e4, the maximum IT was 25 ms, and the normalized collision energy was 30 eV.

### 2.6 Data-Independent Acquisition (DIA) Mass Spectrometry Analysis

The peptides from each sample were analyzed by liquid chromatography-tandem MS operating in the data-independent acquisition (DIA) mode by Shanghai Applied Protein Technology Co., Ltd. (Shanghai, China). Each DIA cycle contained one full MS–selected ion monitoring (SIM) scan, and 30 DIA scans covered a mass range of 350–1800 m/z with the following settings: SIM full scan resolution was 120,000 at 200 m/z, AGC 3e6, maximum IT 50 ms, and profile mode. The DIA scans were set at a resolution of 15,000, AGC target 3e6, maximum IT auto, and normalized collision energy 30 eV. The run time was 120 min with a linear gradient of buffer B (84% acetonitrile and 0.1% formic acid) at a flow rate of 250 nL/min. Quality control samples (pooled sample from an equal aliquot of each sample in the experiment) were injected in DIA mode at the beginning of the MS study and after every six injections throughout the experiment, which were used to monitor the MS performance.

### 2.7 Mass Spectrometry Data Analysis

The raw MS data were analyzed using MaxQuant software (Max Planck Institute of Biochemistry in Martinsried, Germany, version 1.5.3.17). The database was Uniprot_human database, and iRT peptide sequence was added (iRT-Kit; Biognosys, Schlieren, Switzerland). The parameters were set as follows: enzyme was trypsin, max missed cleavages was 2, fixed modification was carbamidomethyl (C), and the dynamic modification was oxidation (M) and acetyl (protein N-terminus). All reported data were based on 99% confidence for protein identification as determined by the false discovery rate (FDR = N(decoy) × 2/(N(decoy) + N(target))) ≤1%). A spectral library was constructed by importing the original raw files and DDA search results into Spectronaut Pulsar X TM_12.0.20491.4 (Biognosys). DIA data were analyzed with SpectronautTM 14.4.200727.47784 search of the above constructed spectral library. Main software parameters were set as follows: retention time prediction type was dynamic iRT, interference on MS2 level correction was enabled, and cross run normalization was enabled. All results were filtered based on a Q value cutoff of 0.01 (equivalent to FDR <1%).

### 2.8 Bioinformatics Analysis 

The DEPs were further analyzed by Gene Ontology (GO) annotation and Kyoto Encyclopedia of Genes and Genomes (KEGG) using DAVID 6.8 database (https://david.ncifcrf.gov/).

### 2.9 Validation of Proteomics Results by ELISA Analysis

The expression of the selected proteins from proteomics was verified by specific human ELISA kits (Abcam, Cambridge, MA, USA), and the experimental steps were performed in duplicate according to the manufacturer’s instructions.

### 2.10 Statistical Analysis

Statistical analyses were performed using SPSS Statistics version 24.0 (SPSS Inc, Chicago, IL, USA). The clinical characteristics of the participants were analyzed using the Student’s *t*-test, chi-square test, or Fisher’s exact test as appropriate. Continuous variables with normal distribution are presented as the mean ± standard deviation (SD) and nominal data are presented as counts and percentages. Receiver-operating characteristic (ROC) curve analysis and calculation of the area under the curves (AUC) were conducted. *p *< 0.05 was considered statistically significant.

## 3. Results

### 3.1 Clinical Characteristics of the Participants

A total of 80 CAC patients and 80 matched-controls were included in this study. Among them, 60 serum samples (30 from CAC patients vs 30 from controls) were collected for proteomic analysis. For the validation set, serum samples from 100 participants, including 50 CAC patients and 50 matched controls, were utilized for ELISA. The clinical characteristics of the participants in the discovery and validation sets are presented in Table 1. There were no significant difference were observed in age, sex distribution, or traditional risk factors between the cases and controls. Consequently, the spectrum of clinical presentations was also well matched between the two groups in either the discovery or validation phase.

**Table 1.** **The clinical characteristics of participants**.

	Screening sample (DIA)			Validation sample (ELISA)		
	Controls	CAC	*p*-value	Controls	CAC	*p*-value
Number	30	30		50	50	
Age (years)	66.07 ± 8.12	68.27 ± 6.71	0.257	65.44 ± 8.46	65.74 ± 8.24	0.858
Male, n (%)	23 (76.67)	24 (80.0)	0.754	31 (62.0)	36 (72.0)	0.288
BMI (kg/$m^{2}$)	25.73 ± 3.25	24.15 ± 3.51	0.075	24.56 ± 3.90	25.65 ± 3.52	0.147
Smoker, n (%)	11 (36.67)	11 (36.67)	1.00	5 (10.0)	8 (16.0)	0.372
Drinking, n (%)	14 (46.67)	8 (26.67)	0.108	12 (24.0)	14 (28.0)	0.648
Diabetes, n (%)	2 ( 6.67)	4 (3.33)	0.389	6 (12.0)	12 (24.0)	0.118
Hypertension, n (%)	20 (66.67)	14 (46.67)	0.118	14 (28.0)	17 (34.0)	0.517
Cholesterol (mmol/L)	4.09 ± 0.96	3.77 ± 1.07	0.218	4.48 ± 1.08	4.10 ± 1.18	0.092
LDL (mmol/L)	2.11 ± 0.66	1.96 ± 0.80	0.426	2.44 ± 0.76	2.19 ± 0.87	0.126
Triglycerides (mmol/L)	1.73 ± 1.14	1.39 ± 0.55	0.141	1.41 ± 0.67	1.24 ± 0.43	0.142
HDL (mmol/L)	1.08 ± 0.18	1.06 ± 0.28	0.832	1.22 ± 0.27	1.13 ± 0.32	0.129
Glucose (mmol/L)	4.87 ± 0.60	5.14 ± 1.33	0.299	4.91 ± 0.71	5.13 ± 1.19	0.266
Clinical presentation, n (%)			0.165			0.840
Stable angina	18 (60.0)	23 (76.67)		21 (42.0)	22 (44.0)	
Unstable angina	7 (23.33)	2 (6.67)		9 (18.0)	10 (20.0)	
NSTEMI	4 (13.33)	2 (6.67)		4 (8.0)	5 (10.0)	
STEMI	2 (6.67)	0 (0)		2 (4.0)	1 (2.0)	

Values are n (%) or mean ± standard deviation (SD). Abbreviations: DIA, data-independent acquisition; ELISA, Enzyme-linked immunosorbent assay; CAC, coronary artery calcification; BMI, Body Mass Index; LDL, low-density cholesterol; HDL, high-density lipoprotein; NSTEMI, non-ST-elevation myocardial infarction; STEMI, ST-elevation myocardial infarction.

### 3.2 Differentially Expressed Proteins Analysis

**Fig. 2.**
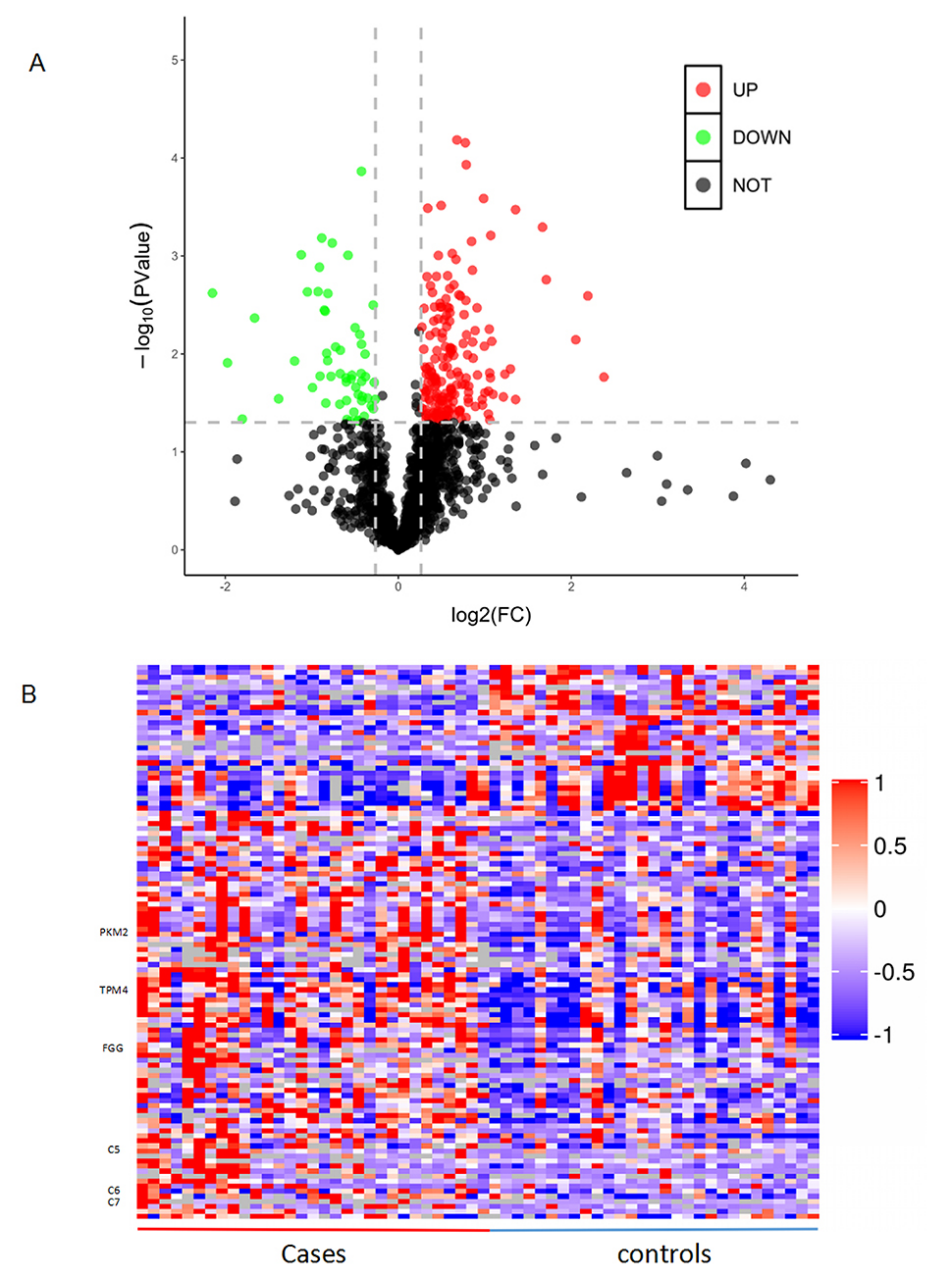
**Differentially expressed proteins between patients with coronary artery calcification (CAC) and controls**. (A) Volcano plot. X-axis represents log2 fold change and Y-axis shows –log10-transformed *p* values. Red dots indicate upregulated proteins, green dots indicate downregulated proteins, and gray dots represent proteins with no significant change. (B) Hierarchical clustering of the 110 dysregulated proteins. Red colour indicates the upregulated protein expression and blue indicates the downregulated protein expression.

A total of 1590 serum proteins were detected between the CAC and non-CAC groups, and the quantitative values for all proteins are shown in **Supplementary Table 1**. Proteins with fold change ≥1.5 or ≤0.66 and *p *< 0.05, and a selected peptide chain >1 were considered as differentially expressed. In total, 110 proteins were found to be differentially expressed between the two groups, of which 81 were upregulated and 29 were downregulated, as illustrated in the volcano plot and by clustering analysis (Fig. 2).

### 3.3 Bioinformatics Analysis of the Differentially Expressed Proteins

**Fig. 3.**
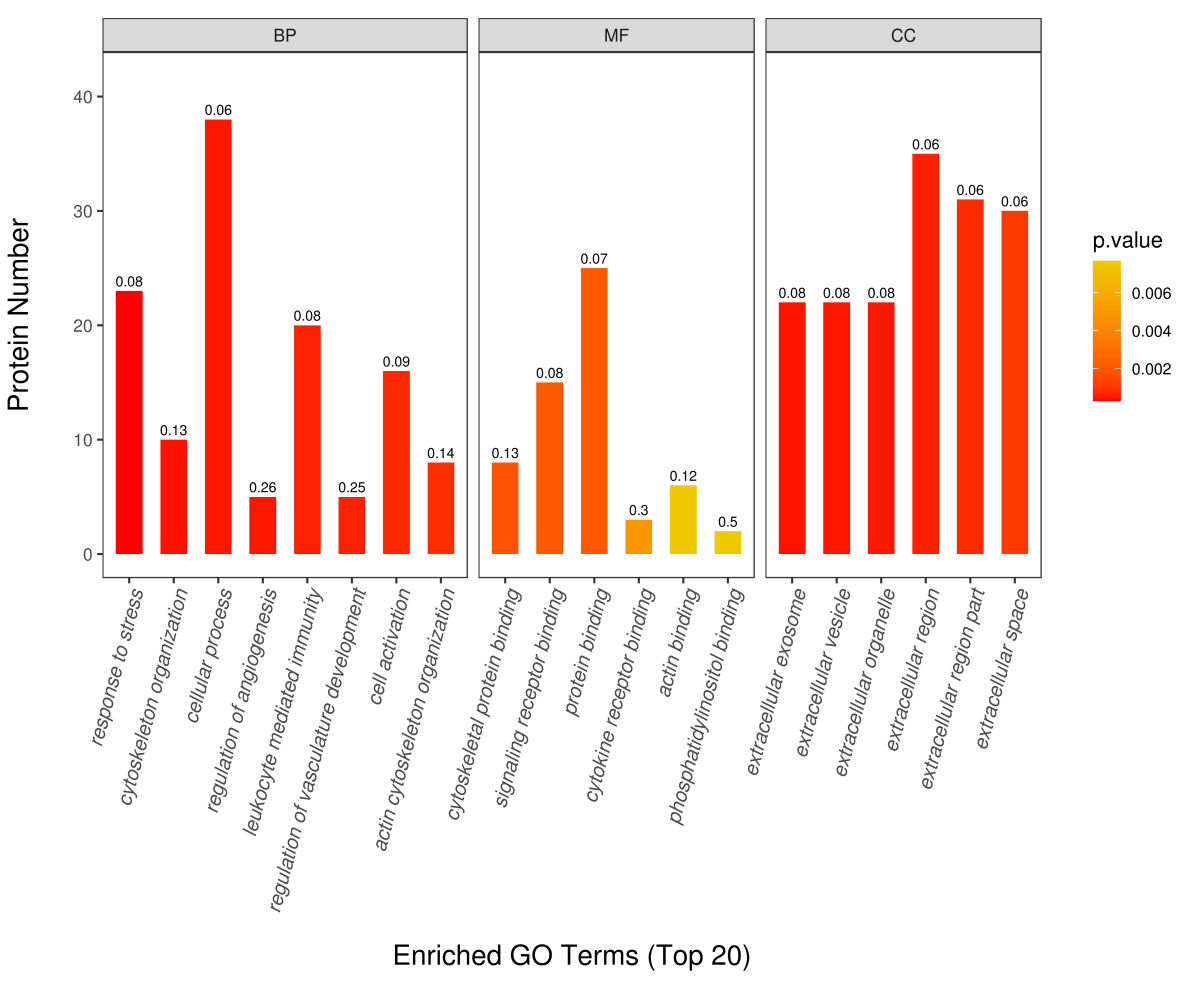
**Gene ontology (GO) analysis of the differentially expressed proteins between patients with coronary artery calcification (CAC) and controls**. GO function consist of biological process (BP), molecular function (MF) and cellular component (CC). The *Y*-axis represents the number of different proteins under each functional classification. The color gradient reflects the size of the *p* value (*p *< 0.05). The label at the top of the bar graph shows rich fator ≤1, and the enrichment factor represents the ratio of the number of differentially expressed proteins (DEPs) over the total number of proteins in each category.

**Fig. 4.**
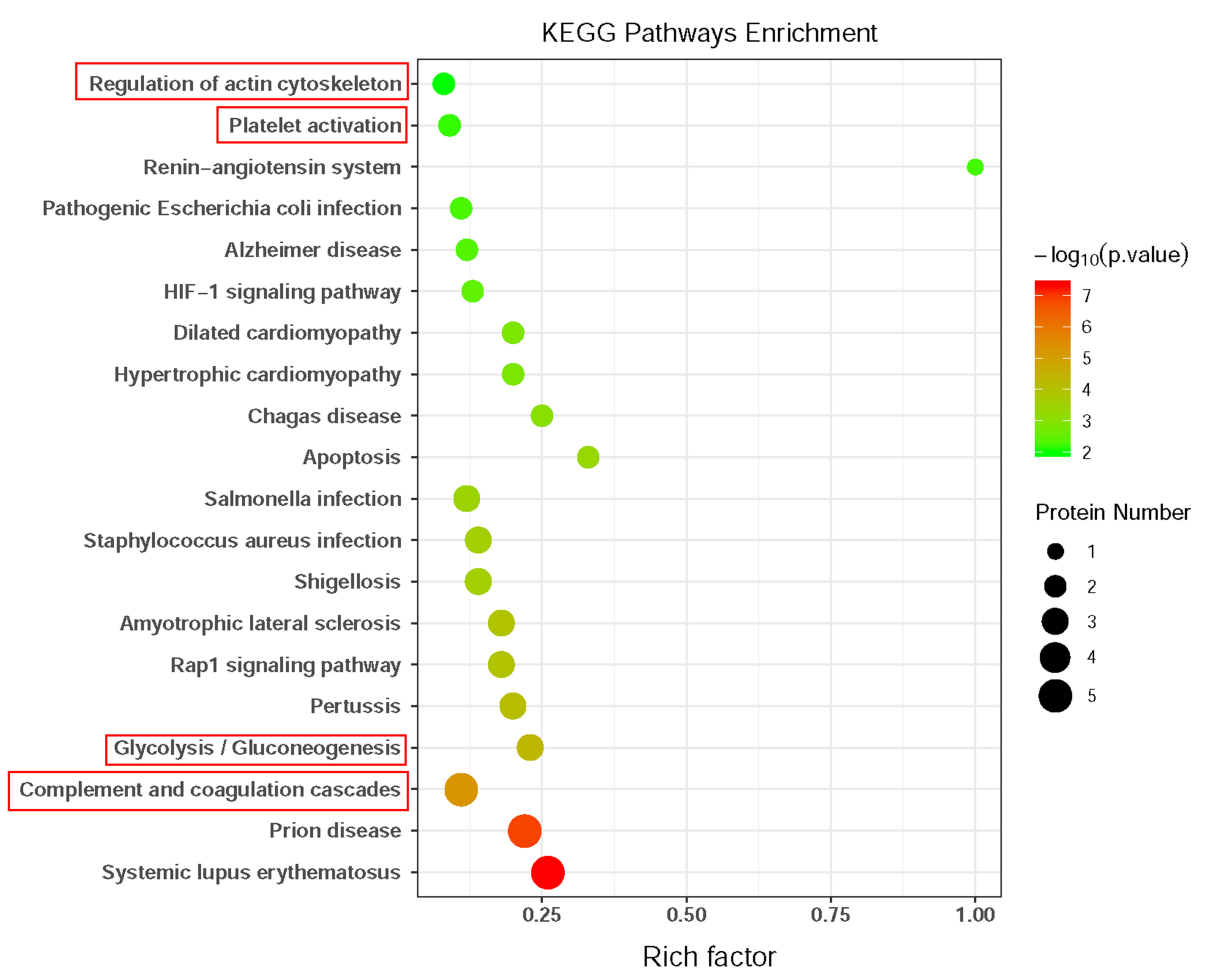
**Kyoto Encyclopedia of Genes and Genomes (KEGG) pathways analysis of the differentially expressed proteins between patients with coronary artery calcification (CAC) and controls**. The bubble color represents significant *p* value, the bubble size represents the number of differentially expressed proteins (DEPs) in the pathway. The *X*-axis is rich factor representing the ratio of DEPs over the total number of proteins in each pathway.

GO and KEGG pathway analyses were performed to investigate the molecular mechanisms and functional activity of DEPs in coronary calcification. Detailed information on the biological process (BP), molecular function (MF), and cellular component (CC) by the GO enrichment analysis is shown in Fig. 3. In the BP category, DEPs were mainly involved in cytoskeleton organization, regulation of vasculature development, and actin cytoskeleton organization. Regarding MF classification, DEPs were associated with actin binding, cytoskeletal protein binding, signaling receptor binding, and cytokine receptor binding. Similarly, CC was mainly localized in the extracellular space, extracellular region, and extracellular vesicle. KEGG pathway results showed that the DEPs were mainly enriched in the complement and coagulation cascades, glycolysis/gluconeogenesis, regulation of the actin cytoskeleton, and platelet activation (Fig. 4).

### 3.4 Validation of Candidate Proteins 

**Fig. 5.**
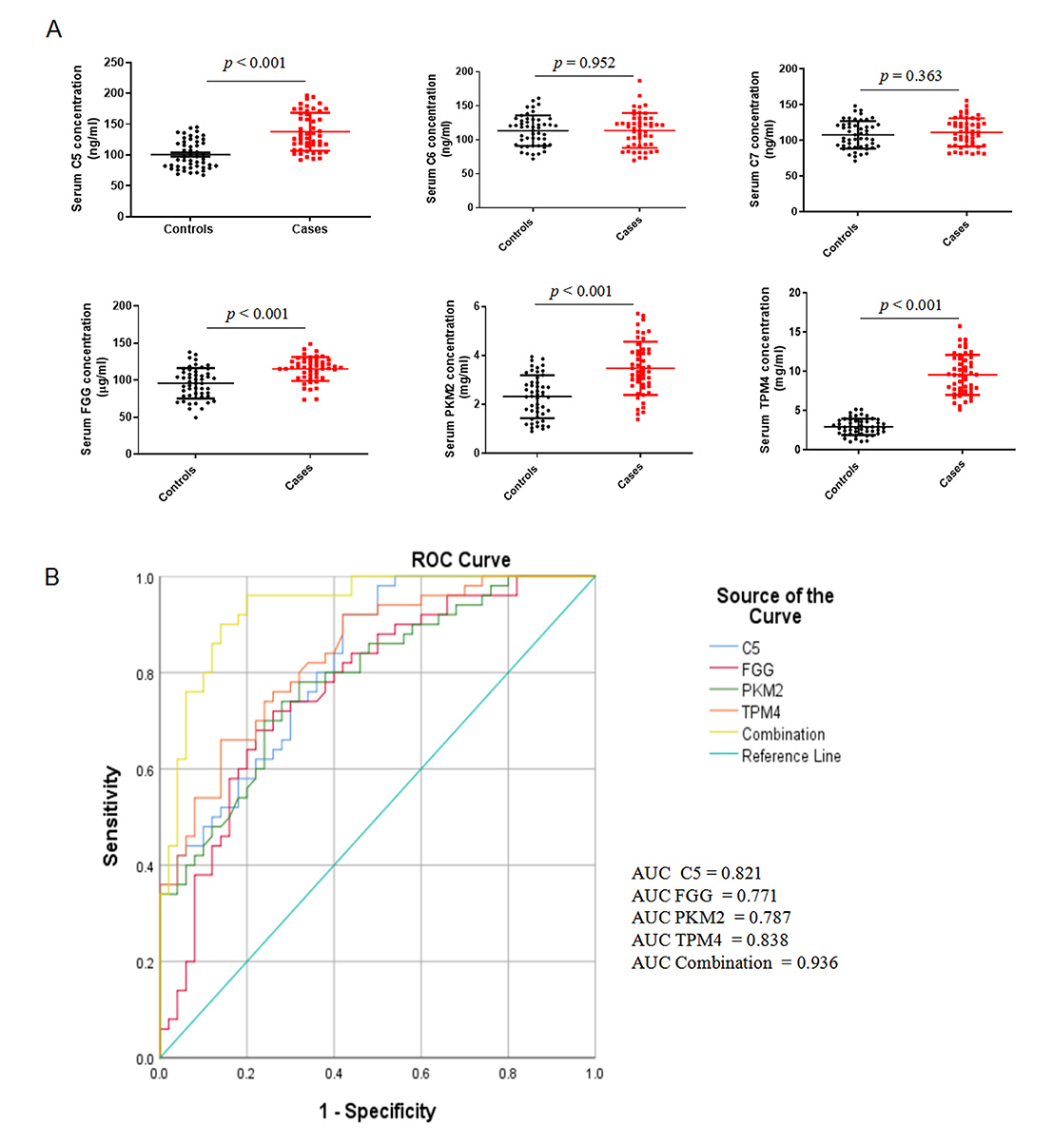
**Validation of candidate serum proteins in an independent cohort**. (A) ELISA validation of six candidate serum proteins (C5, C6, C7, FGG, PKM2, and TPM4) in 50 patients with severe coronary artery calcification and 50 matched controls. Data are presented as mean values ± standard deviation(SD). (B) The diagnostic performances of C5, FGG, PKM, and TPM4 by receiver operating characteristic (ROC) curve analysis.

The validation of candidate protein biomarkers was based on the following criteria: identified proteins containing more than one unique peptide, differential expression fold change ≥1.5, significance based on the standard Student’s *t*-test (*p *< 0.05), quantified proteins corresponding to the gene names were found in the UniProt database, and potential functional or pathological significance in vascular calcification. In this study, KEGG pathway enrichment analysis suggested that complement and coagulation cascade, glycolysis/gluconeogenesis, regulation of actin cytoskeleton, and platelet activation signal pathways are significantly altered in the CAC patients, which are strongly associated with pathological calcification [13, 14, 15]. Because upregulated proteins may represent the disease process and have the potential to be a serum biomarker, the following six upregulated proteins, associated with the pathogenesis of cardiovascular calcification, were chosen for validation by ELISA in independent cohort samples: complement C5 (C5), C6, C7, fibrinogen gamma (FGG), pyruvate kinase isoform M2 (PKM2), and tropomyosin 4 (TPM4).The results demonstrated that serum C5 level in CAC patients was higher than that in non-CAC controls (137.83 ± 30.85 ng/mL vs 100.88 ± 22.95 ng/mL; *p *< 0.001). Serum FGG had the same trend between the two groups (115.18 ± 16.06 μg/mL vs 95.88 ± 20.60 μg/mL; *p *< 0.001). Compared to the non-CAC group (2.32 ± 0.88 mg/mL), the serum level of PKM2 was also significantly increased in the CAC patients group (3.48 ± 1.09 mg/mL; *p *< 0.001). Additionally, patients with CAC showed elevated serum levels of TPM4 in serum compared with the non-CAC cohort (4.48 ± 1.14 mg/mL vs 2.93 ± 1.01 mg/mL; *p *< 0.001). No significant difference in C6, and C7 expression was found between the two groups (Fig. 5A).

ROC curves were calculated to further determine the diagnostic power of serum C5, FGG, PKM, and TPM4 in differentiating CAC patients from non-CAC controls (Fig. 5B). As shown in Table 2, serum C5, FGG, PKM and TPM4 had an optimal AUC >0.75, of which C5 had the largest AUC (0.821 [95% confidence interval: 0.732–0.891]; *p *< 0.001). Moreover, the combination of C5, FGG, PKM and TPM4 had higher AUC, sensitivity, and specificity in discriminating coronary calcification than a single marker alone.

**Table 2.** **Receiver operating characteristics of the four proteins in distinguishing coronary artery calcification from non-calcification controls**.

Proteins	AUC (95% CI)	*p* value	Cut-off ${\mathrm{value}}^{\&}$	Sensitivity	Specificity
C5	0.821 (0.732–0.891)	<0.001	101.68	0.92	0.58
FGG	0.771 (0.676–0.849)	<0.001	103.54	0.68	0.78
PKM2	0.787 (0.700–0.875)	<0.001	2.73	0.78	0.68
TPM4	0.838 (0.751–0.904)	<0.001	3.95	0.66	0.86
C5+FGG+PKM2+TPM4	0.936 (0.869–0.975)	<0.001		0.96	0.80

Abbreviations: AUC, area under curve; CI, confidence interval. Cut-off ${\mathrm{value}}^{\&}$: C5, ng/mL; FGG, μg/mL; PKM2, mg/mL; TPM4, mg/mL.

## 4. Discussion

In this study, we employed a DIA-based quantitative proteomics strategy to explore the differential protein expression in the serum proteome of patients with severe CAC and non-CAC controls. Our results showed that 110 proteins were differentially expressed in both 30 patients with severe CAC and 30 healthy controls, of which 81 proteins were upregulated and 29 proteins were downregulated. KEGG pathway analysis of the DEPs indicated that the complement and coagulation cascade, glycolysis/gluconeogenesis, regulation of actin cytoskeleton, and the platelet activation signaling pathway were significantly changed in the development of coronary calcification.

To validate the proteomic analysis results, the six upregulated proteins with a potential role in coronary calcification were chosen for further study in another independent cohorts of subjects using ELISA. The validated results revealed that the serum levels of C5, FGG, PKM, and TPM4 were significantly increased in patients with CAC as compared to the levels of non-CAC controls. ROC curve analysis also demonstrated that the combined C5, FGG, PKM2, and TPM4 level was better in predicting CAC than use any single marker. To the best of our knowledge, this is the first proteomics analysis of differential protein expression in serum from patients with severe CAC.

This study detected the difference in serum protein expression between the two groups. For the discovery cohort, power calculations for proteomic analysis is the sample size of 60 participants (30 per group) to achieve a power of 80% and an alpha error of 0.05 referring to a previous study [16]. Indeed, the number of patients enrolled in this study does not seem so low when compared to other proteomics studies in coronary artery disease (sample size range from 10 to 20 patients each group) [17, 18, 19].

Accumulating studies provide evidence that innate immune system are key components in triggering cardiovascular calcification through inflammation response [20], whereas complement proteins is a powerful cascade of the innate immunity [21]. Whether complement system activation drives the pathology of vascular calcification has not been adequately addressed. The complement system is usually regulated by the classical, lectin, and alternative pathways. All three pathways merge at the central C3 molecule, leading to the C5 cleavage and a terminal C5b-9 complement complex formation [22]. Recent proteomics analysis identified that complement C5 expression was elevated in calcified aortic valve tissue [23] and subclinical atherosclerotic tissues [24]. Osteogenic transition implicated in the regulation of vascular calcification, and some studies have demonstrated that complement receptor C5 affects osteogenic-mediated vascular calcification by targeting the nuclear factor kappa B transcriptional activity in rodent models [25]. Other studies have revealed that inhibition of C3 and C5 activity can attenuate atherosclerosis [26, 27]. Our study indicates that serum C5 might be a potential biomarker in distinguishing CAC patients from non-CAC individuals, consistent with previous study [24].

Serum FGG levels are associated with prostate cancer and depressed patients [28, 29]. In addition, elevated serum levels of FGG increase the risk of myocardial infarction, which could be explained by platelet aggregation and fibrin formation [30]. Similarly, in this study, serum FGG was found at high levels in CAC patients compared with controls. However, whether FGG plays a causal role in coronary calcification or only acts as a biomarker has remained unclear. Therefore, the role of FGG in the development of coronary calcification needs further investigation.

Pyruvate kinase isoform M2 (PKM2) is a key rate-limiting enzyme for glycolysis and contributes to the vascular smooth muscle cell (VSMC) growth and proliferation during atherosclerosis [31]. Previous studies have shown that PKM2 influences the initiation and progression of atherosclerosis by regulating metabolic reprogramming, immune activation, and tissue inflammation [32, 33]. However, there is limited evidence on the role of PKM2 in vascular calcification. Our study fills the gaps and shows that the expression of serum PKM2 is significantly upregulated in CAC patients. The specific mechanism needs to be clarified in future studies.

Dedifferentiation of VSMCs from the contractile to the synthetic phenotype is a key process in atherosclerosis [34]. TPM4 is associated with several cell functions, such as motility and cytokinesis, is involved in SMC dedifferentiation [35]. TPM4 also contributes to the contractile and synthetic phenotypes of SMCs [36]. Therefore, TPM4 may have pro-atherogenic properties, whereas coronary calcification is strongly correlated with atherosclerosis. Our investigation showed significant upregulation of TPM4 in CAC patients, which suggests that TPM4 plays a critical role in the development of CAC.

## 5. Limitations 

This study had several limitations. First, it was a single-center study with a relatively small number of patients, as DIA technology is costly to apply to many samples. Second, this was a preliminary exploratory study for identifying diagnostic biomarkers for CAC, and more experiments are needed to explore the specific mechanisms of these candidate proteins.

## 6. Conclusions

In summary, our work screened out four proteins (C5, FGG, PKM, and TPM4) were significantly changed in the serum of CAC patients through DIA-based LC-MS/MS combined with ELISA. The pathway of complement and coagulation cascades, glycolysis/gluconeogenesis, regulation of actin cytoskeleton, and platelet activation may participate in the pathogenesis of coronary calcification. ROC curve analysis demonstrated that the combination of C5, FGG, PKM, and TPM4 was well able to distinguish between CAC and non-CAC patients. This work discovered several potential diagnostic markers and provides unique insights into protein expression in vascular calcification.

## Data Availability

The mass spectrometry proteomics data have been deposited to the ProteomeXchange Consortium via the iProX partner repository with the dataset identifier PXD027139 (http://proteomecentral.proteomexchange.org/cgi/GetDataset?ID=PXD027139).

## Supplementary Material

Supplementary material associated with this article can be found, in the online version, at https://doi.org/10.31083/j.rcm2307229.

## References

[1] Lehmann N, Erbel R, Mahabadi AA, Rauwolf M, Möhlenkamp S, Moebus S (2018). Value of Progression of Coronary Artery Calcification for Risk Prediction of Coronary and Cardiovascular Events: Result of the HNR Study (Heinz Nixdorf Recall). *Circulation*.

[2] Mori H, Torii S, Kutyna M, Sakamoto A, Finn AV, Virmani R (2018). Coronary Artery Calcification and its Progression: What Does it Really Mean. *JACC: Cardiovascular Imaging*.

[3] Andrews J, Psaltis PJ, Bartolo BAD, Nicholls SJ, Puri R (2018). Coronary arterial calcification: a review of mechanisms, promoters and imaging. *Trends in Cardiovascular Medicine*.

[4] Lee SJ, Lee IK, Jeon JH (2020). Vascular Calcification-New Insights into Its Mechanism. *International Journal of Molecular Sciences*.

[5] Shenouda R, Vancheri S, Maria Bassi E, Nicoll R, Sobhi M, El Sharkawy E (2021). The relationship between carotid and coronary calcification in patients with coronary artery disease. *Clinical Physiology and Functional Imaging*.

[6] Henein M, Granåsen G, Wiklund U, Schmermund A, Guerci A, Erbel R (2015). High dose and long-term statin therapy accelerate coronary artery calcification. *International Journal of Cardiology*.

[7] Geyer P, Kulak N, Pichler G, Holdt L, Teupser D, Mann M (2016). Plasma Proteome Profiling to Assess Human Health and Disease. *Cell Systems*.

[8] Meyer JG, Schilling B (2017). Clinical applications of quantitative proteomics using targeted and untargeted data-independent acquisition techniques. *Expert Review of Proteomics*.

[9] Krasny L, Huang PH (2021). Data-independent acquisition mass spectrometry (DIA-MS) for proteomic applications in oncology. *Molecular Omics*.

[10] Lee MS, Shlofmitz E, Kaplan B, Alexandru D, Meraj P, Shlofmitz R (2016). Real-World Multicenter Registry of Patients with Severe Coronary Artery Calcification Undergoing Orbital Atherectomy. *Journal of Interventional Cardiology*.

[11] Herrman JP, Azar A, Umans VA, Boersma E, von Es GA, Serruys PW (1996). Inter- and intra-observer variability in the qualitative categorization of coronary angiograms. *The International Journal of Cardiac Imaging*.

[12] Wiśniewski JR, Zougman A, Nagaraj N, Mann M (2009). Universal sample preparation method for proteome analysis. *Nature Methods*.

[13] Demer LL, Tintut Y (2014). Inflammatory, Metabolic, and Genetic Mechanisms of Vascular Calcification. *Arteriosclerosis, Thrombosis, and Vascular Biology*.

[14] Lee K, Kim H, Jeong D (2014). Protein kinase C regulates vascular calcification via cytoskeleton reorganization and osteogenic signaling. *Biochemical and Biophysical Research Communications*.

[15] Bouchareb R, Boulanger M, Tastet L, Mkannez G, Nsaibia MJ, Hadji F (2019). Activated platelets promote an osteogenic programme and the progression of calcific aortic valve stenosis. *European Heart Journal*.

[16] Nyangoma SO, Collins SI, Altman DG, Johnson P, Billingham LJ (2012). Sample Size Calculations for Designing Clinical Proteomic Profiling Studies Using Mass Spectrometry. *Statistical Applications in Genetics and Molecular Biology*.

[17] Krishnan S, Huang J, Lee H, Guerrero A, Berglund L, Anuurad E (2015). Combined High-Density Lipoprotein Proteomic and Glycomic Profiles in Patients at Risk for Coronary Artery Disease. *Journal of Proteome Research*.

[18] Stakhneva EM, Meshcheryakova IA, Demidov EA, Starostin KV, Peltek SE, Voevoda MI (2020). Changes in the proteomic profile of blood serum in coronary atherosclerosis. *Journal of Medical Biochemistry*.

[19] Shin M, Park SH, Mun S, Lee J, Kang HG (2021). Biomarker Discovery of Acute Coronary Syndrome Using Proteomic Approach. *Molecules*.

[20] Passos LSA, Lupieri A, Becker-Greene D, Aikawa E (2020). Innate and adaptive immunity in cardiovascular calcification. *Atherosclerosis*.

[21] Defendi F, Thielens NM, Clavarino G, Cesbron J, Dumestre-Pérard C (2020). The Immunopathology of Complement Proteins and Innate Immunity in Autoimmune Disease. *Clinical Reviews in Allergy and Immunology*.

[22] Rawish E, Sauter M, Sauter R, Nording H, Langer HF (2021). Complement, inflammation and thrombosis. *British Journal of Pharmacology*.

[23] Nagaraj N, Matthews KA, Shields KJ, Barinas-Mitchell E, Budoff MJ, El Khoudary SR (2015). Complement proteins and arterial calcification in middle aged women: Cross-sectional effect of cardiovascular fat. The SWAN Cardiovascular Fat Ancillary Study. *Atherosclerosis*.

[24] Martínez-López D, Roldan-Montero R, García-Marqués F, Nuñez E, Jorge I, Camafeita E (2020). Complement C5 Protein as a Marker of Subclinical Atherosclerosis. *Journal of the American College of Cardiology*.

[25] Kalbasi Anaraki P, Patecki M, Larmann J, Tkachuk S, Jurk K, Haller H (2014). Urokinase Receptor Mediates Osteogenic Differentiation of Mesenchymal Stem Cells and Vascular Calcification via the Complement C5a Receptor. *Stem Cells and Development*.

[26] Yin C, Ackermann S, Ma Z, Mohanta SK, Zhang C, Li Y (2019). ApoE attenuates unresolvable inflammation by complex formation with activated C1q. *Nature Medicine*.

[27] Buono C, Come CE, Witztum JL, Maguire GF, Connelly PW, Carroll M (2002). Influence of C3 Deficiency on Atherosclerosis. *Circulation*.

[28] Peng HH, Wang JN, Xiao LF, Yan M, Chen SP, Wang L (2021). Elevated Serum FGG Levels Prognosticate and Promote the Disease Progression in Prostate Cancer. *Frontiers in Genetics*.

[29] Wang Q, Yu C, Shi S, Su X, Zhang J, Ding Y (2019). An analysis of plasma reveals proteins in the acute phase response pathway to be candidate diagnostic biomarkers for depression. *Psychiatry Research*.

[30] Mannila MN, Lovely RS, Kazmierczak SC, Eriksson P, Samnegård A, Farrell DH (2007). Elevated plasma fibrinogen γ′ concentration is associated with myocardial infarction: effects of variation in fibrinogen genes and environmental factors. *Journal of Thrombosis and Haemostasis*.

[31] Zhao X, Tan F, Cao X, Cao Z, Li B, Shen Z (2020). PKM2-dependent glycolysis promotes the proliferation and migration of vascular smooth muscle cells during atherosclerosis. *Acta Biochimica Et Biophysica Sinica*.

[32] Shirai T, Nazarewicz RR, Wallis BB, Yanes RE, Watanabe R, Hilhorst M (2016). The glycolytic enzyme PKM2 bridges metabolic and inflammatory dysfunction in coronary artery disease. *Journal of Experimental Medicine*.

[33] Lü S, Deng J, Liu H, Liu B, Yang J, Miao Y (2018). PKM2-dependent metabolic reprogramming in CD4+ T cells is crucial for hyperhomocysteinemia-accelerated atherosclerosis. *Journal of Molecular Medicine*.

[34] Allahverdian S, Chaabane C, Boukais K, Francis GA, Bochaton-Piallat M (2018). Smooth muscle cell fate and plasticity in atherosclerosis. *Cardiovascular Research*.

[35] Lin JJ, Eppinga RD, Warren KS, McCrae KR (2008). Human Tropomyosin Isoforms in the Regulation of Cytoskeleton Functions. *Advances in Experimental Medicine and Biology*.

[36] Abouhamed M, Reichenberg S, Robenek H, Plenz G (2003). Tropomyosin 4 expression is enhanced in dedifferentiating smooth muscle cells in vitro and during atherogenesis. *European Journal of Cell Biology*.

